# Exercising with a robotic exoskeleton can improve memory and gait in people with Parkinson’s disease by facilitating progressive exercise intensity

**DOI:** 10.1038/s41598-024-54200-y

**Published:** 2024-02-22

**Authors:** Chris A. McGibbon, Andrew Sexton, Pearl Gryfe

**Affiliations:** 1 Institute of Biomedical Engineering, University of New Brunswick, 25 Dineen Dr, Fredericton, NB E3B 5A3 Canada; 2 Faculty of Kinesiology, University of New Brunswick, Fredericton, NB Canada; 3 Assistive Technology Clinic, 107 Grenadier Cres, Thornhill, ON L4J 7V7 Canada

**Keywords:** Parkinson's disease, Rehabilitation

## Abstract

People with Parkinson’s disease (PwPD) can benefit from progressive high-intensity exercise facilitated with a lower-extremity exoskeleton, but the mechanisms explaining these benefits are unknown. We explored the relationship between exercise intensity progression and memory and gait outcomes in PwPD who performed 8 weeks (2 × per week) of progressive exercise with and without a lower-extremity powered exoskeleton, as the planned exploratory endpoint analysis of an open-label, parallel, pilot randomized controlled trial. Adults 50–85 years old with a confirmed diagnosis of PD participated. Twenty-seven participants randomized to exercise with (Exo = 13) or without (Nxo = 14) the exoskeleton were included in this exploratory endpoint analysis. Detailed exercise logs were kept and actigraphy was used to measure activity count*min^−1^ (ACPM) during all exercise sessions. Only the Exo group were able to progressively increase their ACPM over the entire 8-week intervention, whereas the Nxo group plateaued after 4 weeks. Exercise intensity progression correlated with change in the memory sub-scale of the SCOPA-COG and change in gait endurance from the 6MWT, consistent with the prevailing hypotheses linking high-intensity interval exercise to improved muscle and brain function via angiogenic and neurotrophic mechanisms. Facilitating high-intensity exercise with advanced rehabilitation technology is warranted for improving memory and gait endurance in PwPD.

*Registration*: ClinicalTrials.gov, NCT 03583879 (7/10/2018).

## Introduction

Despite the growing body of evidence^1–6^ supporting exercise as a low-cost and accessible treatment to slow progression of motor and non-motor impairments in people with Parkinson’s disease (PwPD), participation in exercise programs remains a challenge for PwPD^7–10^. High-intensity exercise in particular holds considerable promise as a breakthrough intervention for many health ailments^11–15^ including Parkinson’s disease^1,2^. Although the exercise-induced mechanisms that work to improve body and brain function are becoming better understood in animals and humans^16–18^, the biggest challenge is how to deliver high-intensity exercise interventions to people who can benefit from them but whose condition is a barrier to participating in them.

There are many reasons why PwPD may find it difficult to participate in high-intensity exercise interventions, such as deconditioning, fear of falling, low self-efficacy, low expectations, and decreased motivation^3,19,20^. Solutions are needed for facilitating the delivery of engaging, high-intensity exercise to reduce disability in PwPD ^21^. Examples such as the Park-in-Shape trial^1^—a home-based, gamified, high-intensity aerobic exercise program, and the SPARX trial^2^—a high-intensity treadmill-based walking program, have both shown reduced disease progression on the UPDRS motor III subscale after 6 months of training compared to the control group, but no change in mobility and cognitive functioning was reported over the study period.

Robot-assisted gait training (RAGT) – using stationary treadmill-based robotic systems – offers another potential solution for facilitating gait training interventions in people with disability^22,23^. Although this approach allows for multiple training cycles with controlled levels of assistance, research thus far shows that functional gains are not superior to treadmill training^24–29^. It may be that this type of robot assisted therapy where the machine does all the work may not elicit a high enough metabolic cost to trigger the mechanisms that induce exercise-derived benefits; indeed, the vascular and neuroprotective benefits of exercise requires increasing metabolic demand^18,30^.

Wearable, overground exoskeletons are a broad class of powered orthoses that range from rigid multi-joint actuating robots that can enable standing and walking at limited speed in people with complete paralysis^31–33^, to rigid single-joint actuating exoskeletons^34–37^ to assist with mobility, to single- and multi-joint soft exoskeleton suits that can assist the user dynamically and reduce metabolic demand during walking in people with disability^38–40^. Although each may have their place in the continuum of rehabilitation and restoration, there are few commercial devices suitable for facilitating high-intensity exercise. Such a device must offer dynamic stability, so that users feel safe and confident while using the device during a variety of exercises (ie. beyond stationary walking), while at the same time allowing them to work harder and expend more energy during those exercises than they otherwise would be able to.

The Keeogo Rehab™ exoskeleton, which was used in our study, is one commercial device that has the capability of providing dynamic stability and minimally necessary assistance during common exercise activities such as marching in place, lunges, step ups, walking and stair climbing that can be performed in a controlled environment such as physio gym or clinic. We previously reported^41^ on a randomized controlled trial (RCT) to determine if 8 weeks (2 × per week) of bilateral exoskeleton (Exo) exercise (aerobic, strengthening, and functional) with the Keeogo Rehab™ exoskeleton results in positive changes in cognition, mood, gait, balance and health-related quality of life in adults with PD compared to exercising without an exoskeleton (Nxo) or wait-list control (Con). Participants in the Exo group improved significantly in the memory sub-scale of the SCOPA-COG and in the 6-min walk test (6MWT) following 8 weeks of twice-weekly exoskeleton assisted exercise compared to the Nxo and Con groups.

The planned exploratory endpoint of the trial was quantification of exercise intensity during the therapy sessions via actigraphy in the two exercise groups, and to study its relationship to trial outcomes. Although unavailable at the time of conducting this pilot RCT, Jeng et al.^42^ have recently published a cut-point of 1354 ACPM as the threshold between moderate and high intensity exercise for PwPD, thus allowing us to quantify the degree to which our study participants were able to exercise at an intensity greater than this threshold, and what role, if any, these outcomes play in explaining the observed effects on memory and gait endurance.

As such, this paper explores a mechanistic explanation for the positive changes in memory and gait reported in our main outcomes paper^41^. We specifically focus here on the relationships between exercise intensity and outcomes on the memory & learning sub-scale of the SCOPA-COG and the 6MWT for gait endurance for the two interventions that received exercise: the Exo group (exercise with the exoskeleton) and Nxo (exercised with the exoskeleton). Our prior work^41^ showed that changes in the memory & learning domain of the SCOPA-COG were largely responsible for changes in the overall cognition score, and that the 6MWT was much more responsive than preferred and fast gait walking trials. The following exploratory hypotheses were tested:

1. Did the Exo group achieve a higher exercise intensity (ACPM) during the exercise sessions compared to the Nxo group?
2. Did the Exo group exceed a threshold of 1354 ACPM during exercise sessions proportionally more than the Nxo group?
3. Does progression in exercise intensity correlate with changes in memory and gait outcomes after 8 weeks of twice-weekly exercise?

## Methods

The pilot RCT was registered prospectively at ClinicalTrials.gov (NCT 03583879, 7/10/2018). The study took place at the Assistive Technology Clinic (ATC), Baycrest Hospital, Toronto, Ontario, Canada, between Sept. 2018 and Oct. 2019, and was approved by all relevant Research Ethics Boards (Baycrest, Toronto, ON, and University of New Brunswick, Fredericton, NB). The study was conducted in accordance with the Declaration of Helsinki, and all participants provided signed informed consent prior to enrollment. Details of participants, recruitment, randomization, outcomes measures and interventions are available in the main outcomes paper^41^.

### Assessments of memory and gait

For the present study, based on our preliminary evidence^41^, we focused on relationships between exercise intensity during the intervention and the observed change scores in the “Memory and Learning” sub-scale of the SCOPA-COG, and the 6MWT for gait endurance.

Scales for Outcomes in Parkinson’s-Cognition (SCOPA-COG) is a validated 10 task assessment for quantifying cognitive functioning in PwPD^43,44^. Domains include attention, memory, executive function, delayed recall and visuospatial impairment^45^. The “Memory and Learning” sub-scale has a range of 0–22, where higher scores indicate better cognitive functioning in the memory and learning domain.

The 6MWT (distance in meters) was used to evaluate gait function and endurance^46^, and has been reported as reliable and/or valid in many patient populations^47^. Participants completed the test along a 25 m walkway. The test was repeated three times, and the score was the average of three repetitions. For this test, higher scores (distance walked) indicate better gait endurance.

### Actigraphy during intervention exercises

To date there is no accepted standard for assessing exercise intensity in a clinical setting during the interventions that are being administered. While indirect calorimetry is commonly employed in studies for objectively quantifying pre-post VO2 peak^1,48^, the cart and respirator would make it cumbersome to measure exercise intensity during overground functional exercises and would likely interfere with the delivery of the intervention. Heart rate measures are a common proxy measure of exercise intensity^2,49^, but may not be appropriate for accurate real-time measurement^50^. Actigraphy using small, unobtrusive wearable sensors is a portable and low-cost alternative to indirect calorimetry^42,51,52^, therefore was ideal for the purpose of quantifying exercise intensity while participants performed the exercise sessions.

Participants allocated to Exo or Nxo exercise groups visited the clinic twice-weekly for 8-weeks, for up to a total of 16 sessions. The session activities are listed in Table 1. The Exo group performed all sessions whilst wearing the exoskeleton device (fit and tuned to the user as described elsewhere^41^), whereas the Nxo group performed the exercise sessions in the standard of care fashion, without the exoskeleton. During the first half of the intervention (T1) all participants progressed every week to higher reps/durations and reaching their max capacity, which was then held for the remaining half of the intervention (T2). The purpose of holding the dose mid-way was to enable us to quantify progressive intensity in the 2nd half of the intervention that would be dose independent.

**Table 1:** Exercise program used in the intervention.

**#**	Type	Exercise	Rank	Week 1 2 sessions	Week 2 2 sessions	Week 3 2 sessions	Week 4 2 sessions	Week 5–8 2 sessions
				T1–1st half				T2–2nd half
1	Warm up	Walking warm up	1	5 min	5 min	5 min	5 min	5 min
2	Strength	Squat/ "sit to stands"	6	6 reps × 3 sets	8 reps × 3 sets	10 reps × 3 sets	12 reps × 3 sets	12 reps × 3 sets
3		Static lunges	7	6 reps × 3 sets, per side	8 reps × 3 sets, per side	10 reps × 3 sets, per side	12 reps × 3 sets, per side	12 reps × 3 sets, per side
4		Heel raises	2	6 reps × 3 sets	8 reps × 3 sets	10 reps × 3 sets	12 reps × 3 sets	12 reps × 3 sets
5	Aerobic	Walking HIIT	10	0.5 min fast, 0.5 min slow, 1.0 min rest, 5 sets	1.0 min fast, 1.0 min slow, 1.0 min rest, 4 sets	1.5 min fast, 1.5 min slow, 1.0 min rest, 3 sets	2.0 min fast, 2.0 min slow, 1.0 min rest, 2 sets	2.0 min fast, 2.0 min slow, 0.5 min rest, 3 sets
6	Functional Mobility	Stairs ascent and descent	9	1 flight, 1 min rest	2 flights, 1 min rest	3 flights, 30 s rest	4 flights, 1 min rest	4 flights, 30 s rest
7		Backwards stepping	4	10 reps × 3 sets	12 reps × 3 sets	14 reps × 3 sets	16 reps × 3 sets	16 reps × 3 sets
8		Sidestepping (each direction)	5	10 reps × 3 sets, per side	12 reps × 3 sets, per side	14 reps × 3 sets, per side	16 reps × 3 sets, per side	16 reps × 3 sets
9		Step ups, to step downs (lead w/ each side)	8	6 reps × 3 sets, per side	8 reps × 3 sets, per side	10 reps × 3 sets, per side	12 reps × 3 sets, per side	12 reps × 3 sets, per side
10	Balance	Stride stance	1	10 s × 3 sets, per side	12 s × 3 sets, per side	14 s × 3 sets, per side	16 s × 3 sets, per side	18 s × 3 sets, per side
11		Forward to reverse step, hands free	1	10 reps × 3 sets, per side	12 reps × 3 sets, per side	14 reps × 3 sets, per side	16 reps × 3 sets, per side	16 reps × 3 sets, per side
12		PNF D2 extension in stride stance	3	10 reps × 3 sets, per side	12 reps × 3 sets, per side	14 reps × 3 sets, per side	16 reps × 3 sets, per side	16 reps × 3 sets, per side
13		Downward reach to floor	1	10 reps × 3 sets	12 reps × 3 sets	14 reps × 3 sets	16 reps × 3 sets	16 reps × 3 sets
14		Forward weight shift to upward reach	1	10 reps × 3 sets, per side	12 reps × 3 sets, per side	14 reps × 3 sets, per side	16 reps × 3 sets, per side	16 reps × 3 sets, per side
15		Obstacle course	4	2 min	4 min	5 min	6 min	7 min

*HIIT* High intensity interval training, *PNF D2* Proprioceptive neuromuscular facilitation, “draw sword” shoulder extension.

Detailed exercise logs were kept that tracked each participant’s progress. Dopamine medication timing was also tracked to ensure that all Participants were in the “On” state during intervention sessions.

All participants wore the IMU (GT9X Link, Actigraph Inc., Pensacola, FL) on their right hip during the entire exercise session. The IMU was first wiped of any existing data and then initialized with the participant’s unique ID. The therapist then placed the device on the participant’s hip using a waist-band clip immediately before the session began. After the session the therapist removed the device from the clip holder and immediately docked the device for uploading the data to the Actilife software.

### Actigraphy data processing

After the conclusion of the study the raw files for the 27 participants with up to 16 sessions each were exported as activity count data to csv files and further processed with custom written Matlab algorithms to calculate activity counts per minute (ACPM) by summing the activity counts across the time interval of the session (with leading and trailing noise removed) and dividing by the total time in minutes. Consistent with the recommendation from Jeng et al.^42^ we included only the vertical channel accelerations. ACPM data were further normalized to body mass (ACPM/kg) to account for the mass being moved (ie. the participants’ mass plus the exoskeleton, if applicable).

**Figure 1:**
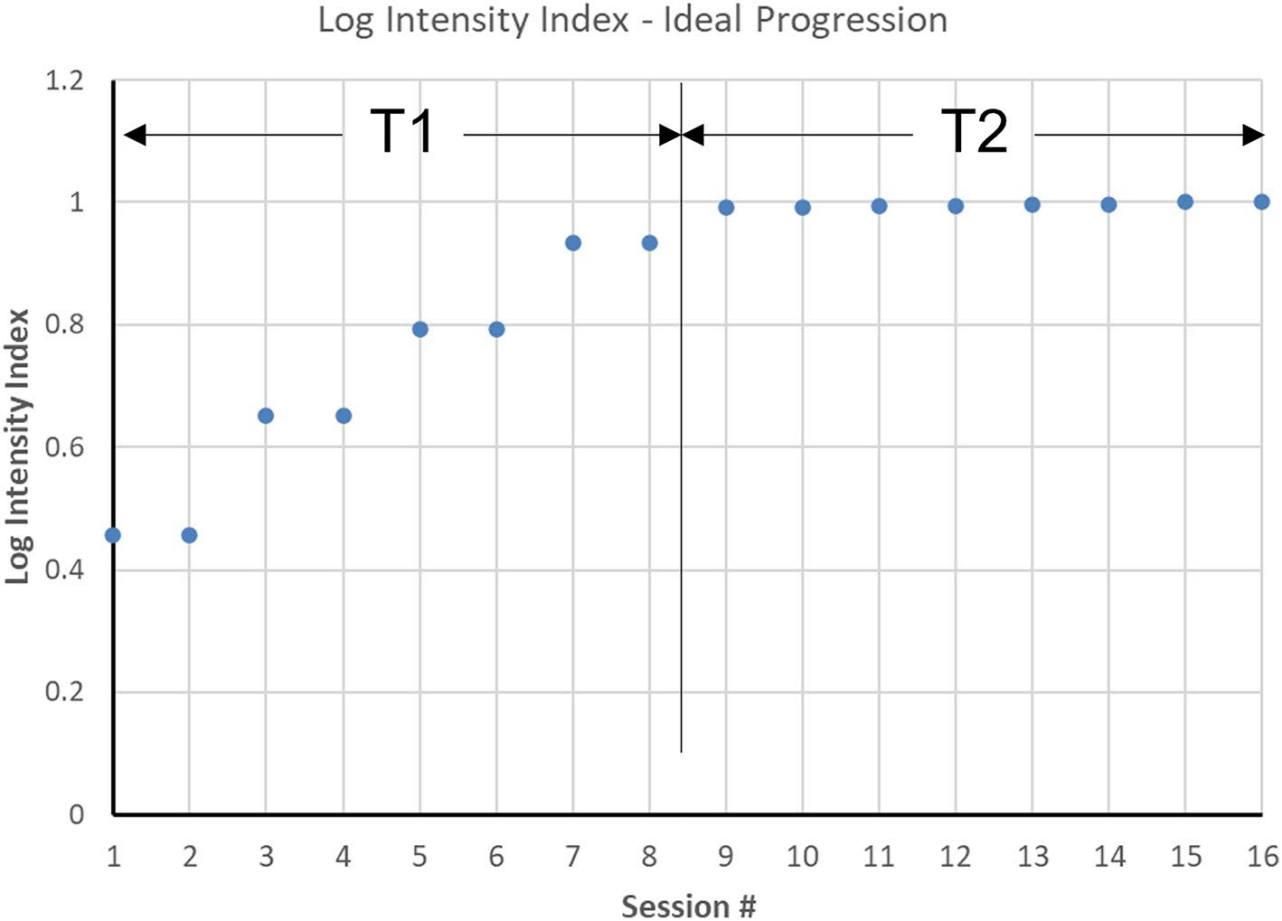
Log intensity index derived from the exercise progression prescription for an ideal hypothetical example where all reps and sets are completed according to the progression schedule. T1 refers to the first half (0–4 weeks) of the intervention which is the progression phase, and T2 refers to the last half (5–8 weeks) of the intervention which is the maintenance phase.

In addition to actigraphy analysis, participants’ exercise logs which contained the actual numbers of sets and intervals for each element of the intervention (Table 1) were transformed into a relative “Log intensity index” for the purpose of comparing how the exercise intervention was delivered to the two treatment groups. We first ranked by consensus all the exercise activities in the intervention by their expected level of vigor (see column 4 of Table 1), multiplied those ranks by the maximum number of minutes, repetitions and sets *planned* for each activity, and summed these to arrive at a prescribed maximum score if the participant performed all activities as scheduled. The algorithm was then applied to each participants’ actual session log data and total scores divided by the prescribed maximum score to arrive at an “index” representing the degree to which the participant achieved the planned dose of exercise in each session. Using the data in Table 1, the prescribed log intensity index was computed and is illustrated in Fig. 1.

### Statistical analysis

To answer the first research question, intensity metrics derived from actigraphy and from the exercise log sheet data were reduced to biweekly averages (for display) and means for intervention intervals T1 and T2, which were then compared between the Exo and Nxo groups using a 2-tailed independent samples t-test.

To answer the second research question, a 2 × 2 Chi-square cross-tabulation analysis was used to compare the frequencies of sessions for Exo and Nxo groups where the participant exceeded the ACPM threshold of 1354^42^.

To answer the third research question, Pearson correlation analysis was conducted to test if there was any relationship between progression in exercise intensity (T2–T1 scores) and memory and gait change scores.

Descriptive statistics were used to describe the sample, which included age, sex, height and weight (as measured in the clinic), body mass index (BMI), Hoehn & Yahr PD disease stage (H&Y), and the Montreal Cognitive Assessment (MoCA).

All statistical analysis were conducted using SPSS (IBM Inc.), with significance set at α = 0.05.

### Ethical approval and informed consent

The study was approved by research ethics boards of Baycrest Hospital, Toronto, Ontario (REB #18-24) and the University of New Brunswick, Fredericton, New Brunswick (REB #2018-136). All participants provided signed informed consent prior to enrollment in the study.

## Results

Data were analyzed from the twenty-seven participants (Exo n = 13, Nxo n = 14) who received exercise interventions. This sample was 69.2 ± 6.7 years of age and consisted of 11 men (41%) and 16 women (59%). Average BMI was 26.6 ± 5.8 kg/m^2^. Participants were distributed across Hoehn & Yahr disease stages I (40.7%), II (29.6%), III (25.9%), and IV (3.7%). MoCA score for the sample was 24.2 ± 3.5 points. These data are shown in Table 2.

**Table 2:** Participants in the exploratory study.

Scale variables	Total		Group A–Exo		Group B–Nxo	
	Mean	SD	Mean	SD	Mean	SD
Age (years)	69.2	6.7	67.6	5.9	70.7	7.3
Weight (kg)	74.0	18.2	71.4	18.4	76.4	18.4
Height (cm)	166.4	8.4	165.4	9.3	167.4	7.7
BMI (kg*m^−2^)	26.6	5.8	26.0	5.8	27.2	5.9
MoCA	24.2	3.5	25.2	3.2	23.2	3.6

Cat. variables	Count	%	Count	%	Count	%
Sex						
Male	11	40.7	4	30.8	7	50.0
Female	16	59.3	9	69.2	7	50.0
H&Y						
Stage I	11	40.7	4	30.8	7	50.0
Stage II	8	29.6	4	30.8	4	28.6
Stage III	7	25.9	5	38.5	2	14.3
Stage IV	1	3.7	0	0.0	1	7.1

*Exo* Exoskeleton exercise training, *Nxo* Exercise training without exoskeleton, *BMI* Body mass index, *MoCA* Montreal Cognitive Assessment, *H&Y* Hoehn & Yahr PD disease stage.

**Figure 2:**
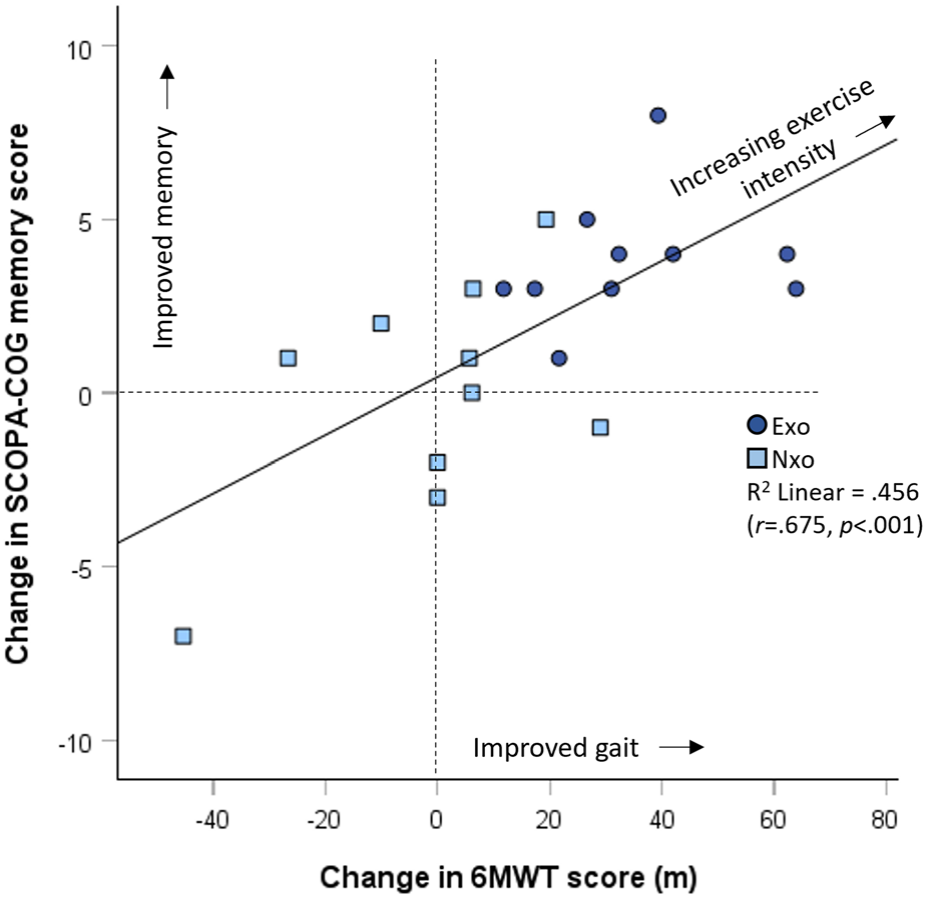
Overlay scatter plot showing the relationship between change in gait endurance as measured by the 6-min walk test (6MWT) and change in memory as measured by the SCOPA-Cog Memory and Learning sub-scale. Light blue boxes represent participants who exercised without the exoskeleton (Nxo) and the dark blue circles represent the participants who exercised with the exoskeleton (Exo). Vertical and horizontal dashed lines demark the zero-change lines.

Memory and gait outcomes were analyzed in our main outcomes paper^41^ across the three treatment groups (Exo, Nxo and Con). Here we show the analysis between just the two exercise groups included in this study (Exo vs Nxo). Memory sub-scale of the SCOPA-COG and 6MWT results are shown Table 3. There were no significant differences (*p* > 0.05) in baseline scores for either measure. Post–pre comparison showed that the Exo group had a significant improvement in memory score (*p* = 0.035) and 6MWT score (*p* < 0.001) compared to the Nxo group, consistent with our previous report that included the waitlist control (Con) group^41^. Also previously unreported was a significant correlation between the 6MWT and SCOPA-COG memory change scores (*r* = 0.675, *p* = 0.001) across the sample of participants in the active exercise interventions (Exo and Nxo), as shown by the overlay scatter plot in Fig. 2.

**Table 3:** Memory and gait scores for trial participants in the exoskeleton exercise group (Exo) and the standard of care exercise group (Nxo).

	Exo	Nxo	Baseline	Post–Pre
	Mean (SD)	Mean (SD)	*p*-value†	
*SCOPA-COG memory*	N = 13	N = 14		
Baseline	8.3 (2.8)	9.4 (3.5)	*.401*	.035*
Post study	11.2 (2.4)	9.7 (3.3)		
Change	2.9 (2.4)	0.4 (3.1)		
*p*-value‡	.001**	*.669*		
*6-Min. Walk Test*	N = 10	N = 11		
Baseline	374.4 (78.9)	369.3 (122.0)	*.911*	< .001*
Post study	409.3 (90.8)	367.9 (126.3)		
Change	34.8 (17.6)	-1.4 (20.4)		
*p*-value‡	< .001**	*.822*		

SCOPA-COG memory = Scales for Outcomes in Parkinson’s-Cognition, memory & learning sub-scale.

^†^Between-groups comparisons: Baseline comparison with one-way ANOVA; Change score comparison controlling for baseline with one-way ANCOVA. *Between-groups change is significant at *p* < .05.

^‡^Within-subjects comparisons: Pre-post comparison with t-test and Bonferroni correction.

### Question 1: Did the Exo group achieve a higher exercise intensity (ACPM) during the exercise sessions compared to the Nxo group?

**Figure 3:**
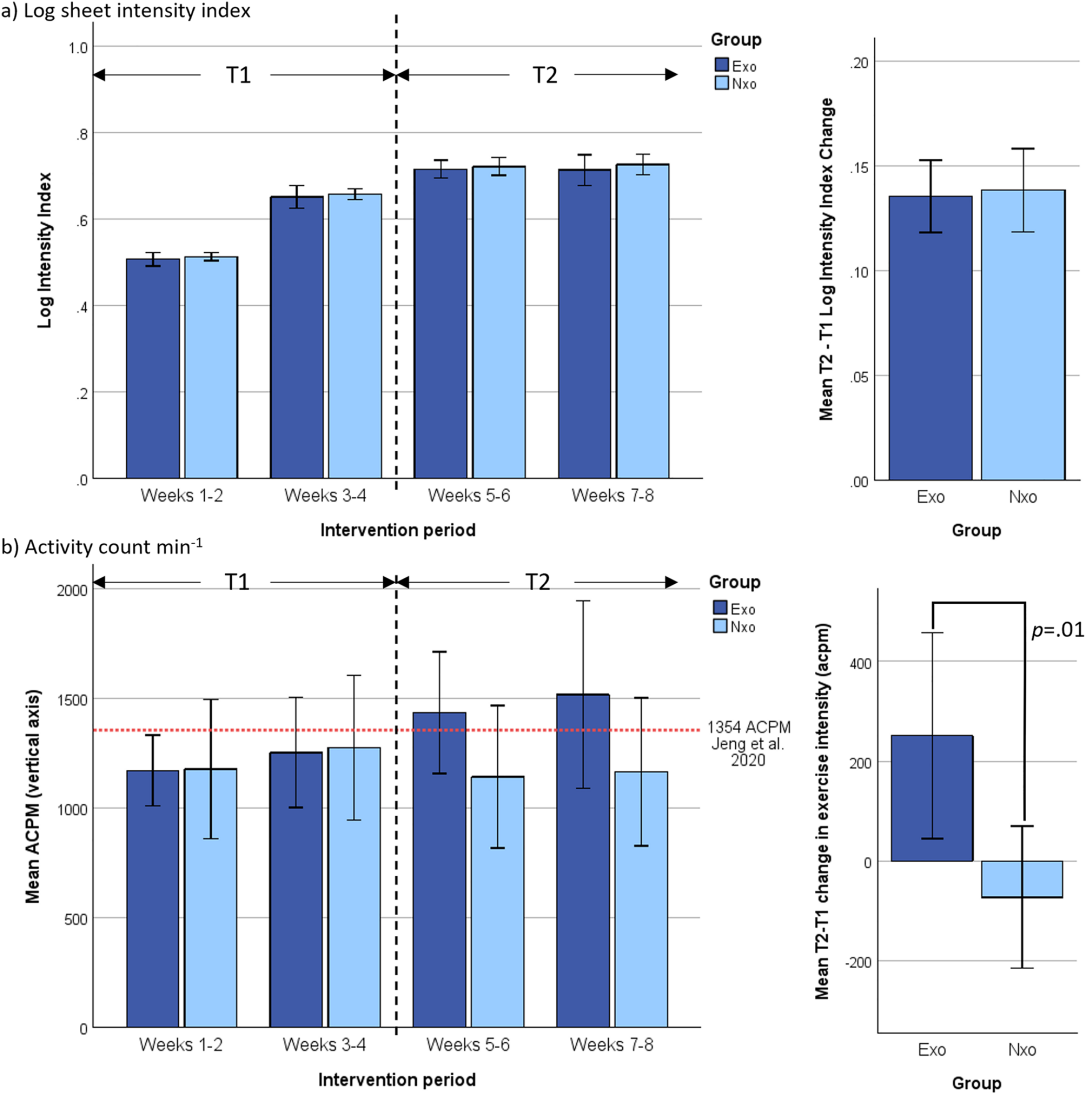
Exercise dose measured two ways: (**a**) Log intensity index showing how well participants were on average able to progress according to the ideal prescription.; and (**b**) Mean activity counts per minute (ACPM) from actigraphy shows how intensely participants were on average able to exercise during the interventions. The left panels show the results averaged over 2-week intervals and the panels on the right show the mean difference between T1 and T2 phases.

Log intensity index from log sheet data (Fig. 3a left) shows that participants in both groups progressed similarly, from about 50% to approximately 75% of the maximal prescribed intensity (ie. no. of reps and duration, etc.). Comparison of the progression from T1 and T2 (Fig. 3a right) between the groups likewise shows that both advanced the same in terms of increasing their sessional activities as prescribed (*p* > 0.05).

In contrast, the actigraphy data (Fig. 3b left) shows that although participants were progressing similarly in prescribed sessional intensity, the activity count rate for the Exo group continued to increase for each bi-weekly interval, whereas exercise intensity for the Nxo group remained relatively unchanged during the trial. Comparison of the progression from T1 and T2 (Fig. 3b right) between the groups shows that Exo group increased their exercise intensity significantly (*p* = 0.01) compared to the Nxo group.

### Question 2: Did the Exo group exceed a threshold of 1354 ACPM during exercise sessions proportionally more than the Nxo group?

As indicated by Fig. 3b (left), only the Exo group’s mean exceeded an exercise intensity above the cut-point threshold of 1354 ACPM, during the T2 period. Analysis of the sessional data showed that in 83 of the 180 total sessions (46%) received by the Exo group, and 64 of the 190 total sessions (34%) received by the Nxo group, participants exceeded the 1354 ACPM threshold during their session. Chi-square analysis showed a significant difference in these proportions (*p* = 0.015) where the Exo group had a higher-than-expected number of sessions that exceeded the 1354 ACPM threshold while the Nxo group had a lower-than-expected number of sessions that exceeded the threshold.

### Question 3: Does progression in exercise intensity (T2-T1) correlate with changes in memory and gait outcomes after 8 weeks of twice-weekly exercise?

**Figure 4:**
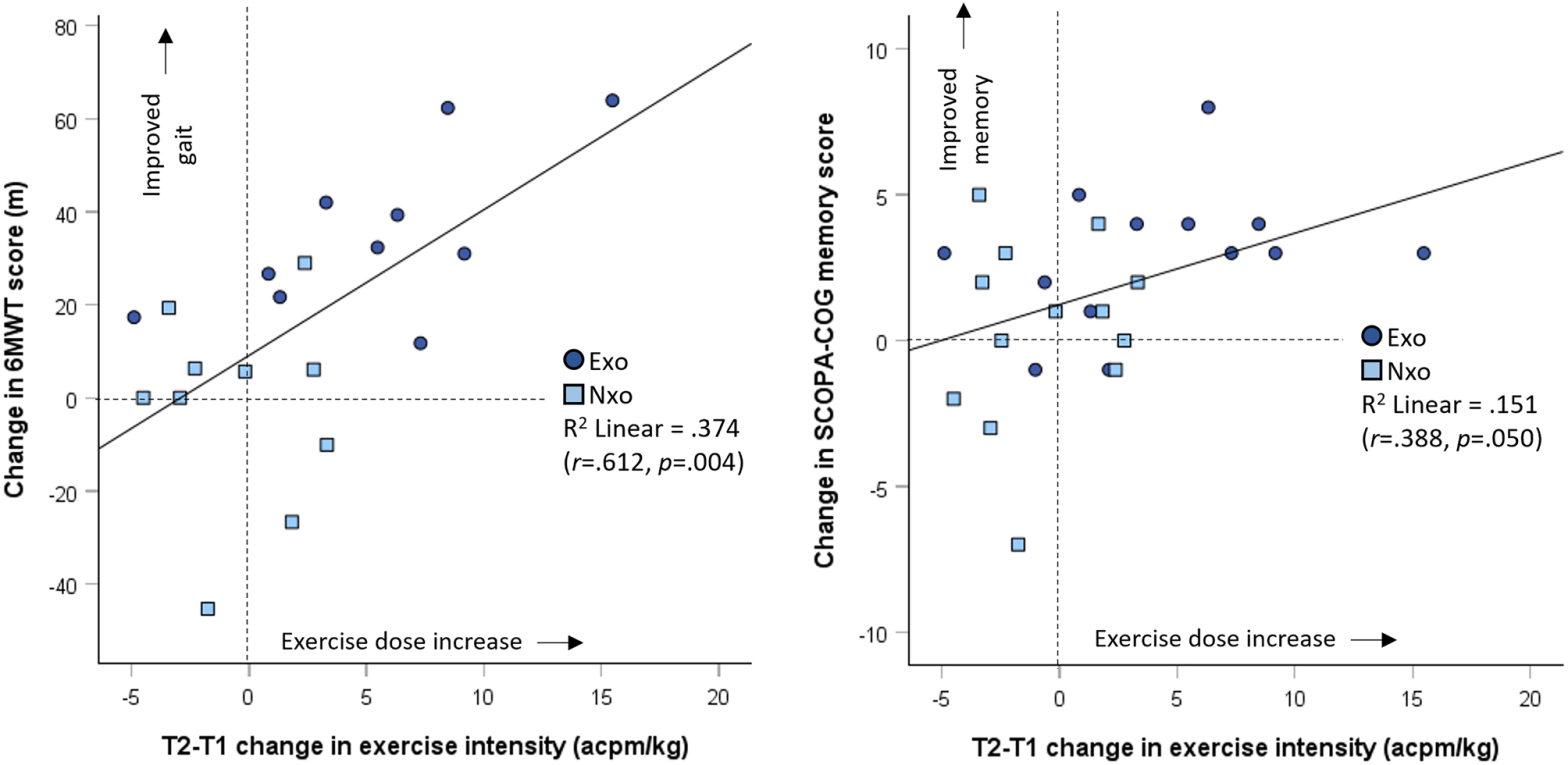
Scatter plots showing the relationship between body mass normalized change in exercise intensity (ACPM/kg) between T1 and T2 phases, with change in gait endurance from 6-min walk test (6MWT) (left panel) and change in SCOPA-Cog Memory and Learning sub-scale score (right panel). Light blue boxes represent participants who exercised without the exoskeleton (Nxo) and the dark blue circles represent the participants who exercised with the exoskeleton (Exo). Vertical and horizontal dashed lines demark the zero-change lines.

Figure 4 shows correlation analysis results of SCOPA-COG memory and 6MWT change scores versus the change in body mass normalized exercise intensity achieved between T1 and T2 of the intervention as measured by actigraphy. Change in exercise intensity between T1 and T2 correlated significantly with change in 6MWT scores and (*r* = 0.612, *p* = 0.004) and change in SCOPA-COG memory score (*r* = 0.388, *p* = 0.050).

Given the significant correlations between change scores in 6MWT and the memory sub-scale of the SCOPA-COG, an ad-hoc partial correlation analysis was conducted to test if the change in exercise intensity was driving this relationship. The partial correlation between 6MWT and SCOPA-COG memory when controlling for ACPM/kg remained significant (*r* = 0.597, *p* = 0.007), meaning that exercise intensity did not explain this effect.

## Discussion

The main objective of this paper was to explore the relationship between exercise intensity and memory and gait outcomes in PwPD who participated in 8 weeks (2 × per week) of progressive functional exercise with and without a lower extremity powered exoskeleton, with the goal of providing a plausible mechanistic explanation for the improvement in memory and gait endurance in the group that exercised with the exoskeleton device, compared to the group that exercised without the exoskeleton^41^.

We demonstrated that PwPD, who have moderate levels of disability, and exercised with the exoskeleton, can feasibly achieve high-intensity exercise (exceed 1354 ACPM) after a relatively short (4 weeks) progression period, and can continue to increase their intensity for at least another 4 weeks, as illustrated in Fig. 3. Achieving those levels of intensity were less frequent with the group that did not wear the exoskeleton, whose intensity levels tended to plateau at moderate-intensity levels. Importantly, our preliminary evidence also suggests that these effects can translate into improvement in important rehabilitation outcomes—memory and gait—which, as illustrated in Figs. 2 and 4, are interconnected. The implications of our findings, and the potential actions they warrant, will next be discussed.

### How can exercising with an overground exoskeleton improve memory and gait?

Past research into robot-assisted gait training (RAGT) systems, which are typically stationary exoskeletons integrated with a treadmill or stepping mechanism, have for the most part failed to show functional gains in PwPD beyond what is achievable using the treadmill alone or other forms of manual gait training therapies for neurological conditions^24–28^. Why then would an overground exoskeleton be expected to yield results different from a stationary one?

If we consider the multi-system integration required for independent human overground locomotion, compared to being walked in a body weight supporting and laterally constraining mechanical apparatus, the non-body weight supporting overground exoskeleton (ie. does not support vertical load) should stress the biological system in ways the stationary robot cannot, which may elicit neurophysiological responses more appropriate for triggering the cascade of mechanisms that can lead to improved muscle and brain function^16–18^.

Our data suggests that exercising with an overground, non-bodyweight supporting, exoskeleton may facilitate achieving this goal by providing movement stability while preserving the need to work against gravity^53^. Because our actigraphy analysis focused on the vertical acceleration counts, we can conclude that participants wearing the device were able to increase their intensity of anti-gravity movements during the exercises, compared to those who exercised without the device.

Our data also show that these effects were not immediate, indicating that one month of progression was required before users of the exoskeleton were able to start achieving consistent exercise intensity levels defined (for the PD population) as “high-intensity”. To elaborate, Fig. 3b (left) provides some key observations of how the participants in our study responded to the two different modes of exercise delivery. During the progression (T1) phase of the intervention both groups exercised at similar sub-threshold intensity levels, and in fact the Nxo group had slightly better (though non-significant) levels of intensity. Participants in the Exo group would have been learning how the exoskeleton interacts with them. After the 4-week progression period (during T2), only the Exo group was able to continue to increase their exercise intensity level. Indeed, the slope of the bi-weekly intensity curve would suggest that, had the intervention been longer, the Exo participants may have continued to increase their levels of intensity beyond the 8-week period.

Indeed, the difference between T2 and T1 mean exercise intensities (in this case, monthly estimates) appeared to be an important indicator of benefits gained from the intervention. Data in Fig. 4 suggests that increasing exercise intensity between the T2 and T1 periods explained about 37% of the variance in 6MWT change scores, and about 15% of the variance in SCOPA-COG memory and learning change scores, when all exercising participants were included. Figure 4 also shows that members of the Exo group appear more frequently in the upper-right quadrant of the scatter plots, again illustrating that wearing the exoskeleton during the exercise interventions had an impact on trial outcomes.

Interestingly, although there was clearly a strong relationship between the 6MWT distance and SCOPA-COG memory and learning change scores, and both were related to T2-T1 ACPM change in exercise intensity, the partial correlation test failed: the relationship observed between change in gait endurance and change in cognition was not moderated by exercise intensity level. This suggests that mobility and cognition are linked via other neurophysiological mechanisms—ie. the mechanism exists despite exercise or exoskeletons—but that exercising at a sufficiently high intensity allows that mechanism to be exploited. In other words, the intensity of the exercise progression determines to some degree where one is positioned on the regression line in Fig. 2. What this means to practice is that it may not be the exoskeleton itself that is explaining the beneficial mechanisms at play, but what the exoskeleton facilitates (ie. novel learning experience plus high intensity interval training) that may be of particular importance to its place in rehabilitation.

More detailed dosing studies are needed that include traditional metabolics, blood biomarkers, and brain imaging, to properly discern how this, and possibly other rehabilitation technologies, interact with proangiogenic and neurotrophic mechanisms that are known to improve muscle and brain function. The role of learning to use a novel robotic technology to improve functioning, and the agency it may bring^54^, is also an area of future inquiry that could provide insights into cognitive benefits of novel training paradigms.

### Research and practice limitations of robots for high-intensity exercise

Recent studies have demonstrated that 6-months of high-intensity treadmill walking (SPARX trial^2^) and similar length home-based exercise using simple, inexpensive, tools and interactive apps (Park-in-Shape trial^1^) can improve clinical (UPDRS) features of PD at long-term follow-up. Such interventions are considerably more accessible than what is proposed here, but it is also important to draw a distinction between the primary endpoints of these long-term studies, which was to slow disease progression in the long-term (as measured by UPDRS), compared to the endpoints of our study which was focused on shorter-term outcomes in mood, memory and gait. These observations are not necessarily at odds, nor are the different approaches incompatible with one another. A possible recommendation stemming from the available data would be short-term high-intensity robot training to “pre-condition” or “top-up” patients prior to and during more accessible and less expensive longer-term interventions.

Nevertheless, overground exoskeletons of the type studied here still represent a highly inaccessible technology, which poses an immediate barrier for translating research like ours into practice. Most importantly, practice guidelines must be developed that are evidence based. Exoskeletons vary in their construction (ie. passive vs. active, rigid vs. soft) and are expensive and difficult to study. In addition, skilled clinicians with expertise in using these devices must be resident in the clinic to conduct research like this, let alone deliver services with the technology. This study is exemplary and perhaps unique in this way, as the research was initiated and driven by practicing therapists at the ATC who had the technology and wanted to know how to best utilize it. Generally speaking, studies like this in advanced rehabilitation technology are rare. Developers need to employ better knowledge translation practices so that their technology’s place in routine clinical practice is driven by the users and recipients.

### Conclusion

We conclude that high-intensity rehabilitation with an overground exoskeleton can translate into improvement in important rehabilitation outcomes, such as memory and gait, in PwPD. The take home message is that technologies suitable for rehabilitation must be able to increase metabolic demand, not reduce it, while at the same time providing enough stability and constraint to build confidence and self-efficacy, that feeds back to engagement and motivation to participate in high intensity exercise. Our data from this study and others suggest that overground powered lower-extremity exoskeletons may possess this capability and could represent a paradigm shift for delivering short, high-intensity functional training programs to PwPD and other movement disorders.

## Data Availability

All data are provided in this manuscript and tables.
